# Neurocognitive Aging Following Acute Illness: Pathobiology and a Framework for Developing Neurotherapeutic Agents

**DOI:** 10.1002/brb3.71542

**Published:** 2026-06-10

**Authors:** Errin Lawrence, Daniel Fulton, Poppy Brown, Subashini Suresh, Mark Morris, Paraskevi Goggolidou, Sree Chaithanya, Marcus Abbawy, Amelia Wild, Prashant Nasa, Niharika A. Duggal, Fang Gao‐Smith, Zubair Ahmed, Suresh Renukappa, Tonny Veenith

**Affiliations:** ^1^ Department of Inflammation and Ageing, School of Infection, Inflammation and Immunology University of Birmingham Edgbaston Birmingham UK; ^2^ Department of Critical Care Medicine and Anaesthesia The Royal Wolverhampton NHS Trust Wolverhampton UK; ^3^ Faculty of Science and Engineering University of Wolverhampton Wolverhampton UK; ^4^ JSS AHER – Wolverhampton Centre For Future Health and Policy Innovation Mysuru India; ^5^ Institute of Acute Care Royal Wolverhampton Hospital and University of Wolverhampton Wolverhampton UK; ^6^ University College London Hospitals NHS Foundation Trust London UK; ^7^ Universtiy of Birmingham Edgbaston Birmingham UK; ^8^ University Hospitals Birmingham NHS Foundation Trust Edgbaston Birmingham UK; ^9^ Dr. B.R. Ambedkar Medical College & Hospital (BRAMC), Bengaluru, Karnataka

**Keywords:** acute illness, cognitive impairment, immune response, neurocognitive ageing, neuroinflammation

## Abstract

**Purpose:**

The purpose of this paper is to synthesize current mechanistic insights and translational progress on neurocognitive aging after critical illness and to outline a framework for developing neurotherapeutic drugs for clinical application.

**Method:**

The method includes a narrative, focused review of clinical studies in patients’ neurocognitive symptoms after critical illness, such as sepsis, trauma, and burns, reported up to December 2025. Evidence was organized across domains, including acute systemic inflammation (ASI), communication channels to the central nervous system (CNS), neuroinflammation and neural integrity, autoimmunity in critically ill patients, and potential therapeutic targets and strategies.

**Finding:**

Acute illness and inflammatory states, including sepsis, trauma, and burns, can lead to accelerated neurocognitive aging, early‐onset cognitive impairment, and memory loss. In acute and critical illness, this is attributed to neuroinflammation, microvascular damage, blood–brain barrier (BBB) disruption, and microglial activation resulting from ASI and immune dysregulation. Current research suggests that it also induces cellular senescence, triggering immune dysregulation and subsequent autoimmunity and autoantibody production, contributing to the progression of neurocognitive aging amid chronic low‐grade inflammation and inflammaging. These processes affect the function and integrity of the CNS, leading to neurocognitive decline.

**Conclusion:**

This review examined the scientific basis for the development of neurocognitive aging after acute illness and how this information may be used to develop potential targets to modulate inflammatory and immune responses and treat this debilitating condition. Such interventions may reduce the burden of senescent cells, mitigate BBB breakdown, restore immune balance, and enhance the brain's neuroplasticity and resilience.

## Graphical abstract

In this review, we discuss the scientific basis for how neurocognitive aging occurs after critical illness, including sepsis, trauma, and burns, and the clinical need to develop therapies to reduce inflammation, protect the blood–brain barrier, remove senescent cells, and enhance neuroplasticity. These strategies aim to prevent cognitive decline and, specifically, memory loss, confusion, and impaired brain function.

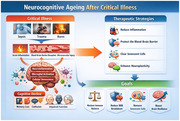

## 1 Introduction

Survival in patients with acute critical illnesses has increased, despite an influx of adults with multimorbidity (Faitna et al. 2024). The resulting physiological insults accelerate aging, with effects that persist for decades. These cognitive, psychological, and physical impairments due to accelerated aging after an acute critical illness are attributed to post‐intensive care syndrome (PICS) (Andonovic et al. 2025). 50%–70% of patients experience a PICS‐related impairment and cognitive dysfunction following a critical illness, even 6 years after discharge, affecting their quality of life (Andonovic et al. 2025; Hopkins and Jackson 2006). Since >20% of the population is projected to be aged 65 and over by 2050, multimorbidity and frailty among survivors are critical. In the US, 20,000 new cases of severe cognitive impairment annually result from severe sepsis and acute illness in over‐65s (Lone et al. 2016).

It is estimated that of the 3 million or so patients who present with sepsis annually, which has a mortality rate of 30%, 59% will show continued slowing of electroencephalogram activity, impaired memory, and hippocampal volume reduction with cognitive and functional impairment, significantly impacting their lives at the one‐year stage (Semmler et al. 2013).

Despite growing recognition of PICS, three key knowledge gaps limit our ability to prevent and treat neurocognitive aging after critical illness. First, how do different inflammatory stimuli engage central nervous system (CNS) aging pathways? Second, the relative contributions of direct neuroinflammation, autoimmune injury, and epigenetic reprogramming to long‐term cognitive outcomes, and, finally, validated neurotherapeutic strategies specifically targeting post‐critical‐illness neurocognitive decline, remain absent from clinical practice.

Acute critical illness does not act in isolation; it accelerates the biology of normal aging and age‐related neurological decline. Chronic inflammation (“inflammaging”) is now recognized as one hallmark of aging, occupying a central position alongside cellular senescence, epigenetic dysregulation, stem cell exhaustion, and altered intercellular communication (Lopez‐Otin et al. 2023). Acute inflammatory insult of critical illness can be understood as an event that transiently amplifies these processes, leaving a lasting impact on brain aging. The mechanisms by which this occurs, and the therapeutic opportunities they create, are the subject of this review.

This review is structured as follows. We start with how acute systemic inflammation (ASI) affects cognitive function and its animal and human evidence, followed by the communication pathways linking peripheral inflammation to the CNS and the neuroinflammatory mechanisms, neural integrity, and individual vulnerability factors.

Finally, we delve into cellular senescence and chronic low‐grade inflammation, the role of autoimmunity, and the roles of current and emerging therapeutic strategies. The aim throughout is to integrate mechanistic insight with translational opportunity, identifying where the evidence is robust and where uncertainty remains. This review addresses these gaps by integrating evidence across molecular, cellular, and clinical domains and by proposing a translational framework for therapeutic development.

### 1.1 Sepsis, Acute Systemic Inflammation (ASI), and a Vulnerable Brain

ASI encompasses systemic immune activation of sufficient magnitude to produce neurobiological signals to the CNS. Sepsis, defined as life‐threatening organ dysfunction caused by a dysregulated host response to infection, is a clinically severe subtype of ASI (Singer et al. 2016). However, major trauma, severe burns, and major surgical procedures also induce ASI through distinct, though partially overlapping, pathobiological mechanisms (Iwashyna et al. 2012). Sepsis‐associated encephalopathy is diffuse brain dysfunction occurring in the setting of sepsis, in the absence of direct CNS infection, primary neurological disease, or other causative factors (Gofton and Young 2012). Hospitalization for severe sepsis is associated with a threefold increase in moderate‐to‐severe cognitive impairment, and analogous neurocognitive sequelae have been documented following major trauma and burns, with a different time course and mechanisms (Iwashyna et al. 2012). Delirium, with an incidence of up to 70% in older patients, is a marker of loss of neuroplasticity in critical illness and is an independent risk factor for neurocognitive impairment and cognitive aging after critical illness (Shafi et al. 2017). While all are affected, older adults and those with preexisting deficits are more likely to experience an abrupt decline in independence, with subsequent reliance on health and social care services (Lone et al. 2016; Pandharipande et al. 2013). Sepsis‐induced inflammation and immune dysregulation, which stay elevated for up to one year after ictus, triggering acute encephalopathy and chronic neurocognitive dysfunction, are poorly understood (Pandharipande et al. 2013; Shafi et al. 2017).

A key question is whether acute critical illness primarily accelerates pre‐existing inflammaging or triggers new neuroimmune cascades, or both, with the relative roles of these processes influenced by patients' biology and the severity of illness. Evidence suggests a dual model. In older, frail individuals with inflammaging, critical illness acts as a “second hit,” amplifying microglial responses and cytokine disruption, leading to faster, exaggerated neuroinflammation (Cunningham 2011; Suk 2026). In younger, resilient patients, illness may trigger independent neuroimmune damage, such as trained immunity reprogramming, blood–brain barrier disruption, and autoantibody production, that persist after the acute phase (Wendeln et al. 2018; Needham et al. 2021). Clarifying this may help precision medicine: targeting upstream inflammation (e.g., senolytics or anti‐inflammatory agents) may help older, vulnerable patients, whereas preventing trained immunity or autoimmunity could benefit patients of all ages. Studies that stratify patients by pre‐illness biological age, inflammaging, and microglial priming are needed to answer these questions and to develop personalized neuroprotective therapies.

**FIGURE 1:**
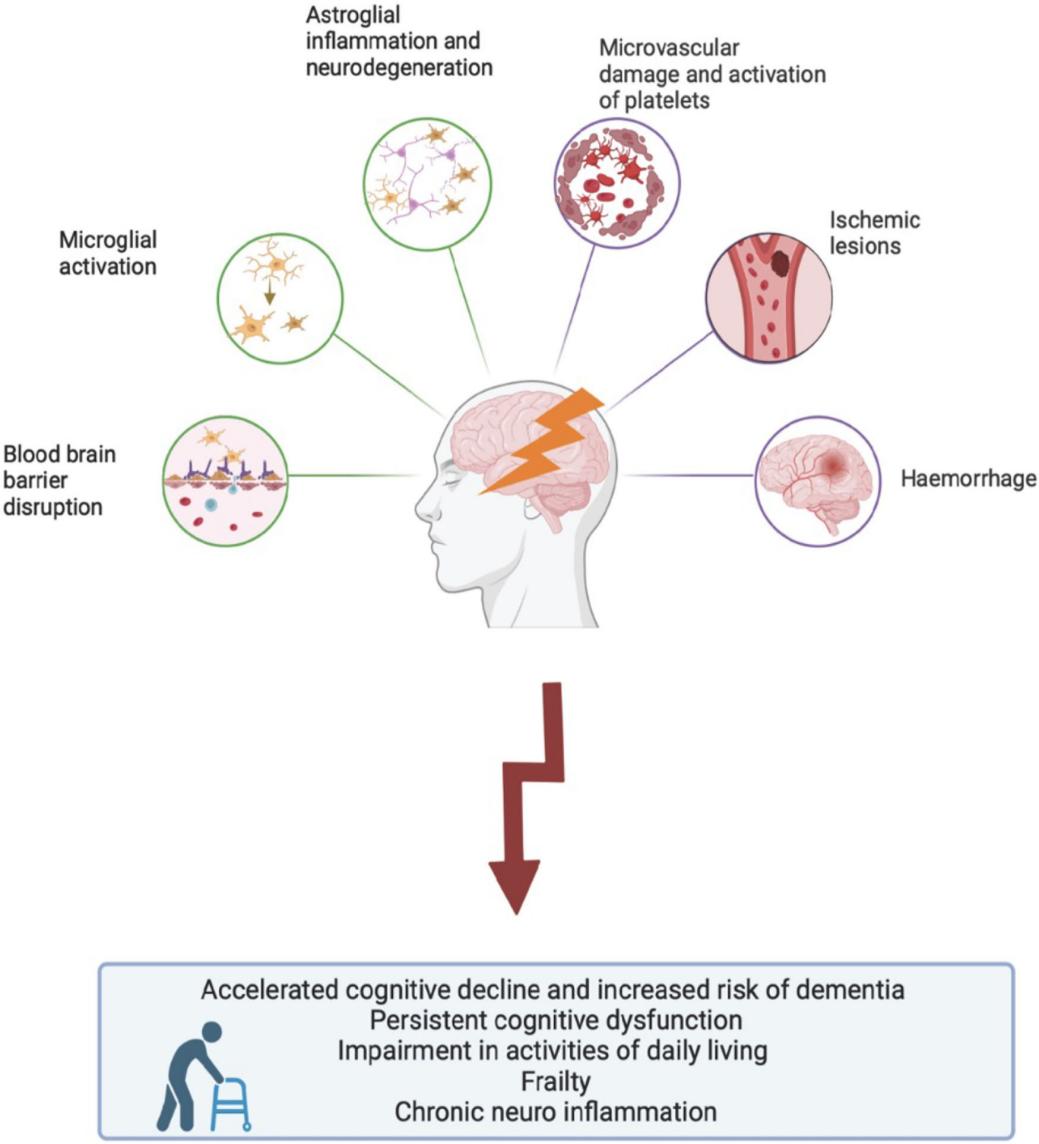
Mechanisms of neurocognitive dysfunction in post‐intensive care syndrome. PICS can lead to blood–brain barrier disruption, microglial activation in the brain, astroglial inflammation and neurodegeneration, microvascular damage, and platelet activation, as well as ischemic lesions in the brain and hemorrhage. All these changes accelerate cognitive decline and increase the risk of dementia, impair the ability to perform daily living tasks, and contribute to frailty. Created with BioRender.com.

Cognitive impairment in septic encephalopathy is often associated with diffuse lesions on neuroimaging, attributable to microvascular damage, neuroinflammation, blood–brain barrier (BBB) disruption, and microglial activation (**Figure** **1**) (Mazeraud et al. 2020). These lesions in sepsis are associated with worse neurocognitive outcomes (Polito et al. 2013).

## 2 ASI and cognitive function

ASI is an immunological mobilization triggered by stimuli such as infections, trauma, or major surgery. ASI releases inflammatory mediators, primarily cytokines and chemokines, by activated immune cells and non‐immune cells such as endothelial and epithelial cells to protect cells and promote their regeneration (Chen et al. 2018). While initially adaptive, ASI often becomes dysregulated and prolonged, leading to widespread tissue damage and organ dysfunction, creating a propagating insult (Ward et al. 2008). These processes trigger inflammatory processes in the brain, at a distance from the initial inflammatory focus (Dantzer et al. 2008). The CNS, being immune‐privileged, is insulated by the BBB from peripheral immune activities, but in ASI, inflammation outside the CNS influences neurocognition, resulting in cognitive impairment (Fitzpatrick et al. 2020).

**FIGURE 2:**
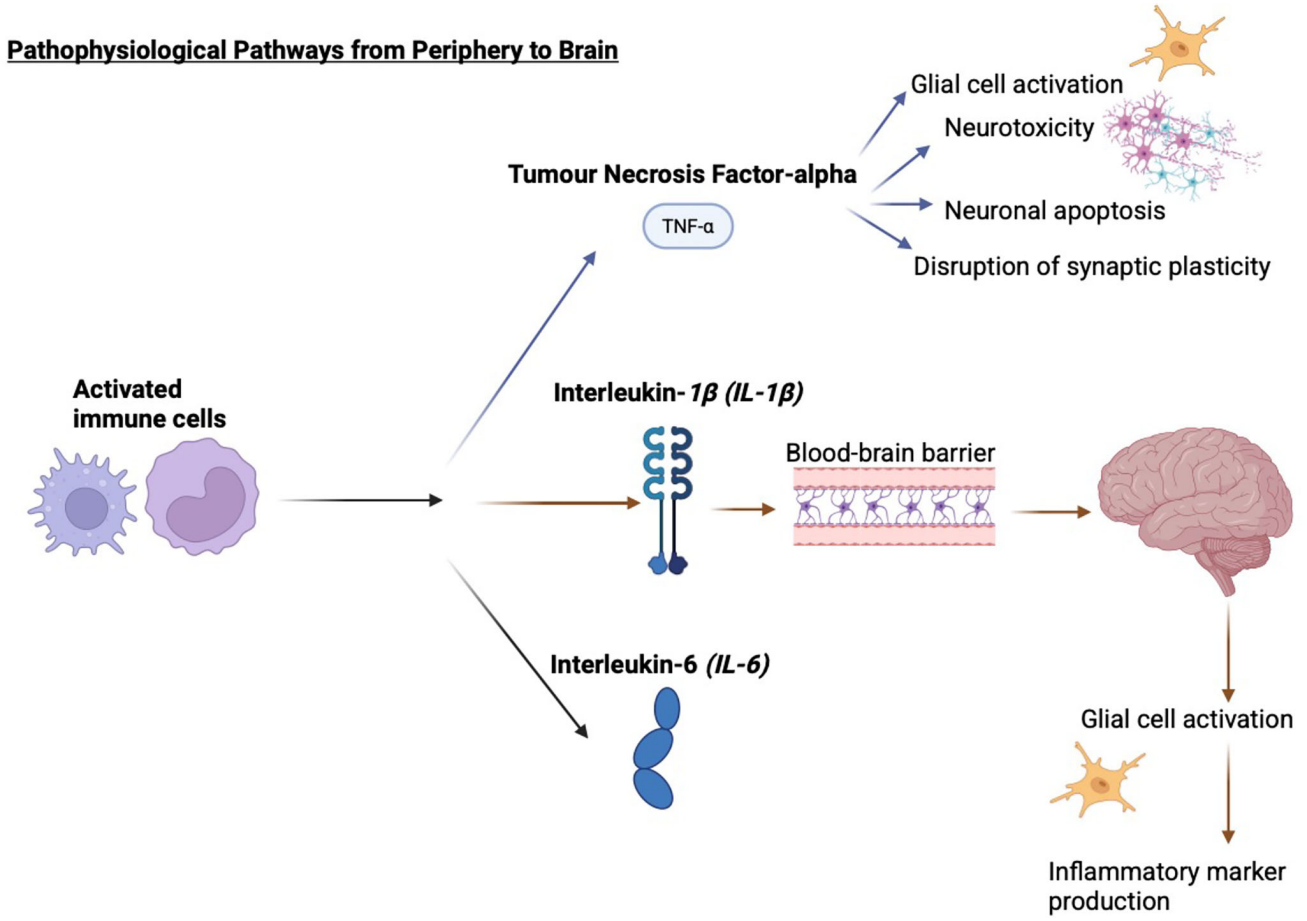
Pathophysiological pathways from periphery to brain. Activation of immune cells leads to the release of TNF‐*α*, IL‐1β, and IL‐6. The downstream effects of TNF‐α include neurotoxicity, neuronal apoptosis, disruption of synaptic plasticity, and glial cell activation. The activation of glial cells contributes to further production of inflammatory markers. IL‐1β crosses the blood–brain barrier to enter the brain and activate glial cells, with the aforementioned downstream effects. Created with BioRender.com.

Induction of ASI using bacterial lipopolysaccharide produced sick behavior and cognitive deficits, particularly in areas that depend on hippocampal function, such as spatial learning and memory (Dantzer et al. 2008). Understanding this is essential for developing targeted strategies in vulnerable patients to improve long‐term outcomes (**Figure** **2**).

Prospective cohort studies indicate that many critical illness survivors, depending on illness severity, age, and follow‐up duration, do not develop lasting cognitive impairment. The type, magnitude, and duration of inflammation influence CNS response, as explained below. Moderate, specific inflammatory activation can temporarily protect the brain, aiding in clearing dysfunctional synapses and debris. The following sections outline key mechanisms through which ASI impairs cognition, noting that outcomes depend on illness severity, brain vulnerability, and biological reserve.

Despite a focus on the periphery‐to‐central spread, this is not a unilateral pathway; it also works from central‐to‐periphery (Brandl and Reindl 2023). The CNS has a key role in maintaining homeostasis and regulating peripheral immunity.

## 3 Communication Channels to the CNS

Peripheral inflammation that transmits signals to the CNS is evolutionarily designed to enable adaptive responses (Quan and Banks 2007). Neurotherapeutic options, hence, need to address pathways, including the circumventricular organs, neural pathways, and cellular pathways. Circumventricular organs, such as the area postrema and median eminence, have fenestrated capillaries lacking the tight junctions of the BBB, enabling interleukin‐1beta (IL‐1β) and tumor necrosis factor‐alpha (TNF‐α) to access neurons and other cells, initiating signaling in the brain parenchyma. This triggers intracellular signaling in the endothelium, leading to the release of secondary inflammatory mediators, such as prostaglandins (e.g., PGE_2_), nitric oxide (NO), and cytokines, which act on adjacent glial cells and neurons (Quan and Banks 2007). The vagus nerve is an afferent pathway for peripheral inflammatory signals from the sites of inflammation, relaying signals to the brainstem nucleus tractus solitarius (NTS) (Borovikova et al. 2000). From the NTS, projections extend to the hypothalamus, the limbic system, and areas involved in autonomic control and the coordination of sickness behavior. This also activates the hypothalamic–pituitary–adrenal axis, a key stress response system. In *Cellular Pathways*, peripheral immune cells, particularly activated monocytes, adhere to cerebral endothelium and transmigrate into the brain parenchyma, releasing additional inflammatory mediators and amplifying neuroinflammation and neuronal dysfunction (McNamara et al. 2023). A permeable BBB allows an influx of plasma proteins, peripheral immune cells, and inflammatory mediators into the brain, triggering a second wave of neuroinflammation by activating resident glial cells (Galea 2021). These communication pathways are summarized in **Table** **1**.

**TABLE 1:** Key inflammatory mediators and their roles in brain communication and neuroinflammation (CVO—Circumventricular organs, BBB – Blood Brain Barrier, LTP – Long‐term potentiation, CCL2 – C‐C Motif Chemokine Ligand 2, CXCL8 – C‐X‐C Motif Chemokine Ligand 8).

Mediator	Primary sources	Key pathways affected	Major effects on CNS
**TNF‐α**	Macrophages, monocytes, T‐cells	Vagal stimulation, circumventricular organs (CVOs), BBB transport, endothelial activation, BBB disruption	Microglial/astrocyte activation, pro‐inflammatory cytokine production, neuronal apoptosis, LTP impairment, synaptic dysfunction
**IL‐1β**	Macrophages, monocytes, endothelial cells	Vagal stimulation, CVOs, BBB transport, endothelial activation, BBB disruption	Microglial/astrocyte activation, sickness behavior, fever, HPA axis activation, LTP impairment, neurotransmitter changes
**IL‐6**	Macrophages, monocytes, T‐cells, endothelial cells	CVOs, BBB transport, and endothelial activation	Microglial/astrocyte activation, acute phase response induction in the brain, potential neurotrophic/neurotoxic effects
**Chemokines (e.g., CCL2, CXCL8)** (Takata et al., 2021)	Macrophages, endothelial cells, astrocytes	BBB disruption (indirectly), act on CNS cells if the BBB is permeable	Leukocyte recruitment (if BBB is permeable), glial activation, and direct neuronal effects
**MMPs (e.g., MMP‐9)** (Quan et al., 1994; Clausen et al., 2020)	Endothelial cells, leukocytes, and glial cells	Primarily act locally at the BBB	Degradation of the BBB extracellular matrix, increased BBB permeability
**Prostaglandins (e.g., PGE2)** (Quan et al., 1994; Clausen et al., 2020)	Endothelial cells (CNS production stimulated by peripheral cytokines)	Act locally within the CNS	Fever, sickness behavior, modulation of synaptic plasticity, sensitization of pain pathways

## 4 Neuroinflammation and Neural Integrity

### 4.1 The Brain Immune Microenvironment

The brain maintains a specialized immune microenvironment in which multiple cellular populations participate in surveillance, homeostasis, and response to injury. Resident microglia, the principal brain‐specific immune cells, continuously survey the parenchyma. Border‐associated macrophages, including perivascular, meningeal, and choroid plexus macrophages, serve distinct functional roles in immune surveillance and antigen presentation (Sun and Jiang 2024). Meningeal lymphatic vessels provide drainage for cerebrospinal fluid (CSF), interstitial fluid, and immune cells, and their dysfunction with aging and acute illness impairs clearance of inflammatory mediators and aggregated proteins from the CNS (Louveau et al. 2015).

Systemic ASI alters this microenvironment. Circulating cytokines and damage‐associated molecular patterns (DAMPs) reach the CNS via the circumventricular organs, disrupted BBB, and neural pathways described in Section 3. The resulting shift in the CNS immune milieu is bidirectional. This bidirectional communication means that the brain is simultaneously a target and a modulator of systemic inflammatory states. An important emerging concept is that ASI can reprogram the CNS immune microenvironment for weeks to months, inducing trained immunity in peripheral monocytes (Wendeln et al. 2018).

### 4.2 Glial Cell Activation

Microglia constantly survey the environment for signs of pathogens, injury, or other disturbances. Peripheral inflammatory signals can trigger microglial activation (Colonna and Butovsky 2017), with morphological and functional changes, transforming into an amoeboid, phagocytic phenotype (Colonna and Butovsky 2017). Activated microglia release additional pro‐inflammatory cytokines such as TNF‐α, IL‐1β, and interleukin‐6 (IL‐6) (Kwon and Koh 2020). Chemokines, reactive oxygen species (ROS), and NO also play a role, creating a self‐perpetuating neuroinflammatory loop within the brain parenchyma (Müller et al. 2025). Traditionally, microglial activation is often referred to as “M1,” a pro‐inflammatory state, and “M2,” an alternative activation state associated with the release of anti‐inflammatory cytokines and neurotrophic factors for tissue repair and debris clearance (Tang and Le 2016). This balance between M1 and M2 phenotypes determines the net neurotoxic or neuroprotective effect (Gao et al. 2023). This historical nomenclature, derived from macrophage biology, is now recognized as a substantial oversimplification. Single‐cell transcriptomic studies have revealed that microglia exist along a continuous spectrum of activation states, with context‐dependent, region‐specific, and disease‐stage‐specific phenotypes that do not map onto M1/M2 designations (Ransohoff 2016). In ASI, microglia may adopt transient pro‐inflammatory phenotypes that shift over time, characterized by impaired homeostatic function, reduced phagocytic efficiency, and persistence of inflammatory signaling, depending on the duration and nature of the original insult (Muzio et al. 2021).

*Astrocyte activation*: Astrocytes, the most abundant glial cells, maintain brain homeostasis, BBB support, neurotransmitter uptake (especially glutamate), ionic homeostasis balance, and metabolic support for neurons (Chen et al. 2023). Astrocytes undergo astrogliosis, characterized by hypertrophy and glial fibrillary acidic protein (GFAP) expression in inflammation, with microglial activation (Lawrence et al. 2023). Microglia also play a key role in inducing astrocyte activation. Microglia activated in disease and aging release a triad of inflammatory mediators (TNF‐alpha, IL‐1alpha, and C1q) that trigger the transition of astrocytes into a neurotoxic “A1” phenotype, whose secretion of soluble factors contributes to neuronal and oligodendrocyte injury (Liddelow et al. 2017). Activated astrocytes also release pro‐inflammatory cytokines to induce neuroinflammation (Chen et al. 2023), while reduced glutamate uptake by dysfunctional astrocytes contributes to excitotoxicity and neuronal damage. Despite their role in promoting neuroinflammation and injury, activated astrocytes are also implicated in anti‐inflammatory and neuroprotective functions. Activated astrocytes release neurotrophic factors, such as brain‐derived neurotrophic factor (BDNF), to promote neuroprotection, but are also responsible the formation of the glial scar, which protects the damaged areas by walling them off, but may impede axonal regeneration by acting as a physical barrier and releasing axon growth inhibitory molecules (Liddelow and Barres 2017; Chiareli et al. 2021). Their involvement in BBB maintenance and repair underscores their role in neuroinflammation, and thus, astrocytesact as both drivers of inflammation and neuronal injury and as key mediators of the repair process (Sofroniew 2015).

### 4.3 Oxidative Stress and Neuronal Dysfunction

Oxidative stress is an imbalance in the production of ROS (e.g., superoxide anion, hydroxyl radicals, and reactive nitrogen species (RNS) such as NO and peroxynitrite) and the brain's capacity to detoxify these reactive intermediates (Ienco et al. 2011). During neuroinflammation, activated microglia and astrocytes and dysfunctional mitochondria become significant sources of ROS and RNS (Obrador et al. 2020). While necessary for host defense, these molecules can cause collateral damage to neurons when overproduced, with a loss of neuronal integrity and function (Cerejeira et al. 2010).

ROS and RNS can damage all major classes of cellular macromolecules, impairing neuronal synaptic plasticity, which is crucial for learning and memory, and triggering neuronal death by

- Lipid peroxidation (Ayala et al. 2014).
- Protein carbonylation and nitration leading to protein misfolding, aggregation, and loss of function for enzymes, receptors, and structural proteins (Berlett and Stadtman 1997; Xiang et al. 2013).
- Deoxyribonucleic acid (DNA) damage causing mutations and strand breaks, potentially leading to neuronal dysfunction or apoptosis by triggering the p53‐p21 pathway (Dash et al. 2025; Filomeni et al. 2015).

### 4.4 Alterations in Neurotransmission and Synaptic Plasticity

The neuroinflammatory cascade disrupts multiple neurotransmitter systems essential for normal cognitive operations, as follows:

*The cholinergic system is* essential for attention, learning, and memory (Reale and Costantini 2021). ASI reduces acetylcholine release and alters the function of cholinergic receptors (Reale and Costantini 2021). This cholinergic deficit, along with the anticholinergic load, is strongly implicated in the attentional impairments and delirium in acute illnesses (van Gool et al. 2010).

*Dopaminergic pathways*: Dopaminergic pathways in mesolimbic and mesocortical systems are crucial for motivation, reward processing, executive functions, and working memory. Inflammatory cytokines alter dopamine synthesis, release, and reuptake, leading to dysregulated dopaminergic signaling (Felger and Miller 2012). This underlies anhedonia and motivational deficits, which are core components of sick behavior and may impair working memory and executive control (Felger and Treadway 2017).

*Serotonergic pathways, originating from the raphe nuclei*: Inflammation can affect serotonin metabolism by shunting its precursor, tryptophan, down the kynurenine pathway (Dantzer et al. 2008), leading to reduced serotonin availability and the production of neuroactive kynurenine metabolites. These alterations can contribute to depression and anxiety‐like symptoms, which often co‐occur with cognitive dysfunction (Dantzer et al. 2008).

*Glutamate receptors*: Glutamate, an excitatory neurotransmitter, is vital for synaptic plasticity and cognition, but excessive activation leads to excitotoxicity and neuronal death. Neuroinflammation disrupts glutamate homeostasis through impaired astrocytic uptake or excessive release from damaged cells or glia (Haroon et al. 2017). Pro‐inflammatory cytokines can also directly modulate glutamate receptor function.

### 4.5 Synaptic Plasticity

Beyond dysregulation of the global neurotransmitter system, neuroinflammation directly impacts synaptic plasticity, learning, and memory (Mottahedin et al. 2017), including long‐term potentiation (LTP), a long‐lasting enhancement of synaptic transmission following high‐frequency stimulation, which is studied in cellular learning and memory models of the hippocampus. Pro‐inflammatory cytokines, notably IL‐1β and TNF‐α, impair LTP in in vivo hippocampal slices (Vereker et al. 2000).

*Synaptic structure and receptor trafficking*: TNF‐α can influence synaptic structure and the trafficking of glutamate receptors (e.g., α‐amino‐3‐hydroxy‐5‐methyl‐4‐isoxazolepropionic acid receptors) to and from the synapse, which is critical for its strength and plasticity (Zipp et al. 2023).

This involvement of multiple neurotransmitter systems explains the diverse cognitive and behavioral changes observed during ASI, including deficits in attention, memory, executive functions, mood, and motivation.

### 4.6 Selective Vulnerability

The brain regions show selective vulnerability, with some more affected than others. Regions such as the hippocampus and prefrontal cortex (PFC), and associated neurochemical systems such as cholinergic and dopaminergic pathways, are more vulnerable and may represent novel neurotherapeutic targets (Pandya and Patani 2021). Attention, which is selective concentration amid multiple stimuli, is disrupted by AIS, manifesting as inattention (e.g., vigilance), difficulty focusing, and cognitive deterioration, leading to an inability to multitask (Balter et al. 2019; Mekhora et al. 2024). The underlying mechanisms involve the impact of pro‐inflammatory cytokines, especially IL6 and TNF‐*α*, on PFC function and associated fronto‐parietal attention networks, with a disruption of cholinergic pathways, critical for arousal and attention (Brosnan et al. 2022). Learning and memory processes, particularly those dependent on the hippocampus and medial temporal lobe structures, are significantly affected by ASI (Krabbe et al. 2005). This is true for declarative memory, which involves the conscious recall of facts (semantic memory) and events (episodic memory). This impacts memory consolidation, where recently acquired labile memory traces are stabilized and transformed into permanent storage (Schapiro et al. 2019). Clinically, patients with AIS may be able to recall events from their distant past but struggle to learn new information or to remember events during or shortly after the illness (Kuhlmann et al. 2005). Executive functions, an umbrella term for higher‐order cognitive processes, are compromised during ASI (Schapiro et al. 2019). These impaired functions of the PFC and its connections include planning, working memory, cognitive flexibility (set‐shifting), and decision‐making (Gross and Grossman 2010). Studies have shown that peripheral inflammation can impair performance on tasks requiring working memory updating and cognitive flexibility (Harrison et al. 2009). The frontal‐striatal circuits mediating these functions are known to be sensitive to inflammatory insults, including alterations in dopaminergic neurotransmission and direct effects of cytokines (Engler‐Chiurazzi et al. 2023).

The characteristic pattern of cognitive impairment, affecting attention, memory consolidation, and executive functions, mirrors common clinical syndromes such as delirium and post‐operative cognitive dysfunction (POCD) (Rudolph et al. 2008). An understanding of this profile is essential for selecting appropriate neuropsychological assessment tools for both research and clinical practice, as detailed in Table 2.

**TABLE 2:** Cognitive domains affected by ASI and tasks.

Cognitive domain	Findings with ASI	Neuropsychological assessment tasks	Implicated brain regions and pathways
**Sustained attention** (Balter et al. 2019; Riccio et al. 2002)	Reduced accuracy/speed on vigilance tasks, increased omission errors	Continuous performance test, Sustained attention to response task	Prefrontal cortex, fronto‐parietal networks, cholinergic pathways
**Selective attention** (Christensen et al. 2011)	Increased interference from distractors, difficulty filtering irrelevant information	Stroop test, Flanker task, Dichotic listening task	Anterior cingulate cortex, prefrontal cortex
**Divided attention** (Langeard et al. 2021)	Impaired performance when managing multiple tasks or information streams simultaneously	Dual‐task paradigms, Trail making test Part B	Prefrontal cortex, fronto‐parietal networks
**Processing speed** (Brydon et al. 2008)	General slowing of reaction times and task completion	Digit symbol substitution test, Simple reaction time tasks, Trail making test Part A	Diffuse cortical and subcortical networks, white matter integrity
**Working memory** (Miller et al. 2009)	Reduced capacity to hold and manipulate information (e.g., mental arithmetic, sequencing)	Digit span backwards, n‐Back task, letter‐number sequencing	Dorsolateral prefrontal cortex, fronto‐parietal networks
**Episodic memory (consolidation)** (Savage 1992)	Impaired delayed recall of verbal or visual material, relatively intact immediate recall	Rey auditory verbal learning test – delayed recall, California verbal learning test – delayed recall, logical memory (WMS) – delayed recall	Hippocampus, medial temporal lobes, Papez circuit
**Executive function (planning)** (Bollen et al. 2017; Zook et al. 2004)	Difficulty formulating strategies, organizing steps, and foresight	Tower of London, Tower of Hanoi, Maze tests	Dorsolateral prefrontal cortex, Fronto‐striatal circuits
**Executive function (flexibility)** (Bollen et al. 2017; Kortte et al. 2002)	Increased perseverative errors, difficulty shifting cognitive set, or adapting to new rules	Wisconsin card sorting test, Trail making test Part B (TMT‐B)	Ventrolateral prefrontal cortex, orbitofrontal cortex, fronto‐striatal circuits
**Executive function (inhibition)** (Bollen et al. 2017)	Difficulty suppressing prepotent responses	Go/No‐Go task, Stroop test (inhibition component)	Inferior frontal gyrus, pre‐supplementary motor area

Sickness behavior due to inflammation is marked by lethargy, malaise, low appetite and thirst, social withdrawal, anhedonia, somnolence, and depressive or anxiety symptoms. It includes cognitive changes like poor concentration, mental fatigue, and reduced motivation. It is triggered by pro‐inflammatory cytokines such as IL‐1β, TNF‐α, and IL‐6, which affect brain regions regulating mood, motivation, and arousal (McFarland et al. 2021). This adaptive response conserves energy and limits social contact, helping the body recover from infection and reduce further risks. If the inflammatory trigger is severe or prolonged, sickness behavior can transition from an adaptive strategy to a maladaptive state, significantly impacting the quality of life and functional capacity.

### 4.7 Delirium and Postoperative Cognitive Dysfunction (POCD)

Delirium is an acute neuropsychiatric syndrome characterized by a disturbance in attention and awareness, accompanied by changes in cognition or perceptual disturbances (e.g., hallucinations) that develop over a short period (hours to days) and fluctuate in severity in the elderly and acutely ill (Stollings et al. 2021). ASI, arising from conditions such as sepsis, major surgery, or severe trauma, is a major precipitating factor for delirium. The key mechanisms include all the characteristics described before (Fong and Inouye 2022; McQuail et al. 2015; Wilson et al. 2020).

POCD is defined as a decline in mental function, typically affecting domains such as memory, attention, and executive function, which can be detected in patients following major surgery, particularly when compared to their pre‐operative baseline. In POCD, transient cognitive changes can occur in the immediate postoperative period, and cognitive deficits can persist for weeks, months, or even longer. POCD is common in elderly patients undergoing major procedures like cardiac, orthopedic, or abdominal surgery. Compelling evidence implicates ASI in surgical trauma, releasing pro‐inflammatory cytokines like IL‐6 and TNF‐*α*, linked to POCD development and severity. These signals likely cause neuroinflammation, BBB dysfunction, and neuronal disruption, leading to cognitive impairments (Evered et al. 2018). POCD can have significant long‐term consequences, including reduced quality of life, impaired functional independence, delayed return to work, and, in some studies, an association with increased long‐term mortality (Suraarunsumrit et al. 2024).

***Individual patient susceptibility***: The cognitive effects of ASI vary among individuals. A complex interplay of intrinsic and extrinsic factors influences vulnerability to cognitive dysfunction following inflammation. This “multi‐hit” model views ASI as an acute “hit,” but the brain's resilience and response depend heavily on pre‐existing conditions and traits (Marzola et al. 2023). Advancing age is a non‐modifiable risk factor for experiencing more severe and prolonged cognitive deficits following ASI (Marzola et al. 2023). The aging brain exhibits several age‐related changes, including inflammaging, compromised BBB integrity, microglial senescence, reduced neurotrophic support, and diminished cognitive reserve.

“Inflammaging” is a chronic, low‐grade, sterile inflammatory state often seen in healthy aging. This pro‐inflammatory environment can “prime” the immune system, including CNS microglia, causing an exaggerated and poorly regulated neuroinflammatory response during ASI (Franceschi and Campisi 2014; Krabbe et al. 2004). The BBB may become more permeable with age, even without acute pathology, making the aging brain more susceptible to the influx of peripheral inflammatory mediators (Elwood et al. 2017). Microglia in the aging brain can adopt a senescent or “primed” phenotype. These primed microglia exhibit an exaggerated pro‐inflammatory response to stimuli and may be less efficient in their neuroprotective and reparative functions (Wendimu and Hooks 2022). The production of neurotrophic factors, which support neuronal survival and plasticity, may decline with age, reducing the brain's capacity for resilience and repair. Cognitive reserve refers to the brain's ability to buffer the effects of neuropathology and maintain function. While not directly an immune factor, older individuals may have accumulated age‐related neuropathology (e.g., subclinical cerebrovascular disease and early amyloid deposition) that reduces their cognitive reserve, making them more susceptible to clinically apparent cognitive decline, with an ASI (Jones et al. 2006).

### 4.8 Preexisting Illness

***Neurodegenerative diseases***: Individuals with pre‐existing neurodegenerative conditions such as Alzheimer's disease (AD) or Parkinson's disease (PD) often harbor ongoing neuroinflammation and “primed” microglia (Badanjak et al. 2021; Neher and Cunningham 2019). A peripheral inflammatory stimulus can act as a “second hit,” amplifying neuroinflammatory processes and accelerating cognitive decline. ASI has been shown to worsen AD pathology, for example, by increasing amyloid‐beta production and tau hyperphosphorylation (Holmes et al. 2009).

***Metabolic syndrome, obesity, diabetes, cardiovascular, and cerebrovascular disease***: These are conditions characterized by chronic, low‐grade ASI and endothelial dysfunction. This baseline inflammatory state can increase vulnerability to the effects of an acute inflammatory episode, potentially leading to a more robust neuroinflammatory response and greater cognitive impairment (Monteiro and Azevedo 2010). Conditions affecting vascular health can compromise cerebral blood flow and BBB integrity, making the brain more vulnerable to hypoxic–ischemic damage and inflammatory insults.

***Chronic infections***: Persistent infections, such as HIV, chronic viral hepatitis, or even chronic periodontitis, can maintain systemic immune activation, priming the CNS response after ASI.

***Genetic factors***: An individual's genetic makeup plays a role in determining their susceptibility to inflammation‐induced cognitive dysfunction. Variants in genes that encode for key components of the immune response can influence the magnitude, duration, and nature of both systemic and central inflammatory responses (Bales 2000).

***Genetics***: In particular, polymorphisms in genes encoding for pro‐inflammatory cytokines (e.g., TNF‐α, IL‐1β, IL‐6) and anti‐inflammatory cytokines (e.g., interleukin‐10 [IL‐10]), as well as their receptors, have been associated with differential susceptibility to inflammatory diseases and their cognitive sequelae (Fujita et al. 2019). Specific TNF‐α gene variants may lead to higher TNF‐α production in response to a stimulus, potentially increasing the risk of more severe neuroinflammation and cognitive deficits (Bartolato et al. 2015). The APOE ε4 allele, a well‐established genetic risk factor for AD, has also been linked to poorer cognitive outcomes after ASI (e.g., surgery, infection, and traumatic brain injury). The mechanisms that require further investigation may point toward interactions between APOE ε4, neuroinflammation, and BBB function (Di Battista et al. 2016).

Variants in genes related to innate immune receptors (e.g., Toll‐like receptors), complement factors, or enzymes involved in inflammatory mediator synthesis could also contribute to individual differences in immunological responses (Newton and Dixit 2012). It could be speculated that genetic factors influencing the structural integrity of the BBB or the intrinsic resilience of neurons to inflammatory or oxidative stress may also modulate cognitive outcomes after ASI, and although the links remain elusive, further investigations will shed light on the exact effect of genetic variants on these outcomes.

***Epigenetics and biological brain aging***: Epigenetic modifications without changes in the genome, such as DNA methylation (DNAm), histone modifications, and non‐coding RNA regulation, are altered during acute inflammation. This provides a mechanistic explanation for individuals with similar genetic profiles with varying neurocognitive outcomes following critical illness (Cheyne et al. 2026).

Epigenetic clocks, based on blood and tissue DNAm patterns, can provide a quantitative estimate of biological age acceleration associated with critical illness and systemic inflammatory states (Dugue et al. 2018;). The GrimAge2 clock, which uses plasma protein methylation surrogates alongside DNAm data, shows accelerated aging in ICU survivors compared with age‐matched controls (Lu et al. 2022; Cohen et al. 2022).

Importantly, tissue‐specific brain epigenetic clocks, including the CorticalClock, diverge from blood‐based estimates in neuroimmune conditions, suggesting that the brain ages differentially from the rest of the body following acute illness (Shireby et al. 2022). Histone modifications driven by inflammatory mediators are also mechanistically relevant. Pro‐inflammatory cytokines, particularly IL‐6 and TNF‐alpha, alter histone deacetylase activity in microglia and neurons, shifting the balance toward transcriptional repression of neuroprotective and anti‐inflammatory gene programs (Pinheiro et al. 2025).

MicroRNA (miRNA) regulation is another epigenetic layer of relevance. miR‐146a and miR‐155 are key regulators of the innate immune response in microglia, and their dysregulation after sepsis has been shown to sustain a pro‐inflammatory microglial phenotype beyond the resolution of the acute illness (Bswas and Lopez‐Collazo 2009). Post‐sepsis epigenetic “imprinting” of immune cells, well characterized in monocytes, may have a CNS‐specific analogue in trained microglial responses (Mildner et al. 2024). Lifestyle and environmental exposures that modify epigenetics, including prior infection burden, nutritional status, physical activity, smoking, and psychosocial stress, offer an explanation for inter‐individual variation in neurocognitive vulnerability (Chakif and Furrer 2026).

***Baseline cognitive reserve and brain health***: Cognitive reserve is the brain's ability to withstand damage while keeping cognitive function, often indicated by education, job complexity, and mental activities. Those with higher reserves may better compensate for neural damage from ASI, experiencing fewer or less severe symptoms. Conversely, those with lower reserves may reach cognitive impairment faster when faced with an inflammation challenge (Pettigrew and Soldan 2019). Pre‐existing brain health, independent of neurodegenerative diseases, also influences outcomes. For instance, individuals with brain atrophy, white matter lesions, or a history of a vulnerable brain may have reduced neuroplasticity, making them less tolerant to ASI‐induced neuroinflammation and prone to cognitive deficits. Identifying these factors is vital for risk stratification and tailoring therapies to reduce neurocognitive dysfunction.

### 4.9 Mechanisms of Chronic Critical Illness (CCI)

A total of 49% of patients with surgical critical illness develop chronic critical illness (CCI), and these patients remain in the hospital three times as long (Stortz et al. 2018). 80% of CCI patients have poor outcomes, such as long‐term care, nursing homes, or hospice. Additionally, 37% die within six months of sepsis diagnosis. In the US, only 20% go home, 30% die in hospital, and one‐year survival is under 50% (Mira et al. 2017). CCI is characterized by persistent organ dysfunction for ≥14 days with a cytokine storm, affecting organs like the kidneys, brain, and muscles. CCI features maladaptive immunity, chronic inflammation, immunosuppression, and protein breakdown, leading to PICS (**Table** **3**). Immune issues stem from reduced natural killer activity, impaired neutrophil phagocytosis, and low lymphocyte counts due to T and B cell apoptosis, leading to a higher neutrophil/lymphocyte ratio. These changes raise the risk of secondary bacterial and viral reactivations (Duggal et al. 2018; Fenner et al. 2021; Vanzant et al. 2014). Patients who develop CCI have a robust early inflammatory response with sustained elevations in IL‐6, IL‐8, and IL‐10 and persistence of immune suppression with elevated sPDL‐1 levels (Stortz et al. 2018). There is a direct correlation between muscle wasting and inflammation, which is more pronounced with multiple organ failure than single organ failure (Puthucheary et al. 2013). The hormonal stress response in critical illness follows a biphasic pattern, with the acute phase directing the organism to a catabolic state to survive the acute insult. The second phase is maladaptive, hampering recovery effectively (Téblick et al. 2019).

**TABLE 3:** Phenotypes of CCI.

Phenotype	
Persistent inflammation	Persistently elevated inflammatory cytokines IL‐6, IL‐8, and IL‐10 Persistently elevated CRP Reduced albumin Ongoing damage resulting in DAMPs Elevated soluble programmed death ligand‐1 (sPDL‐1)
Immune suppression	Myeloid‐derived suppressor cell (MDSC) expansion Increased secondary bacterial infections and reactivation of viral infections Lymphopenia Weak phagocytosis
Protein catabolism	Loss of muscle mass Weight loss Reduced physical function and independence Elevated mean urinary 3‐MH to creatinine ratio (muscle catabolism) Low levels of insulin‐like growth factor binding protein‐3 (IGFBP) (reduced anabolic signaling)
Endocrine dysfunction	Reduced pulsatile secretion of GH Reduced insulin‐like growth factor beta Reduced TSH, T4, and T3 secretion Reduced ACTH adrenal gland atrophy Hyperglycemia

**FIGURE 3:**
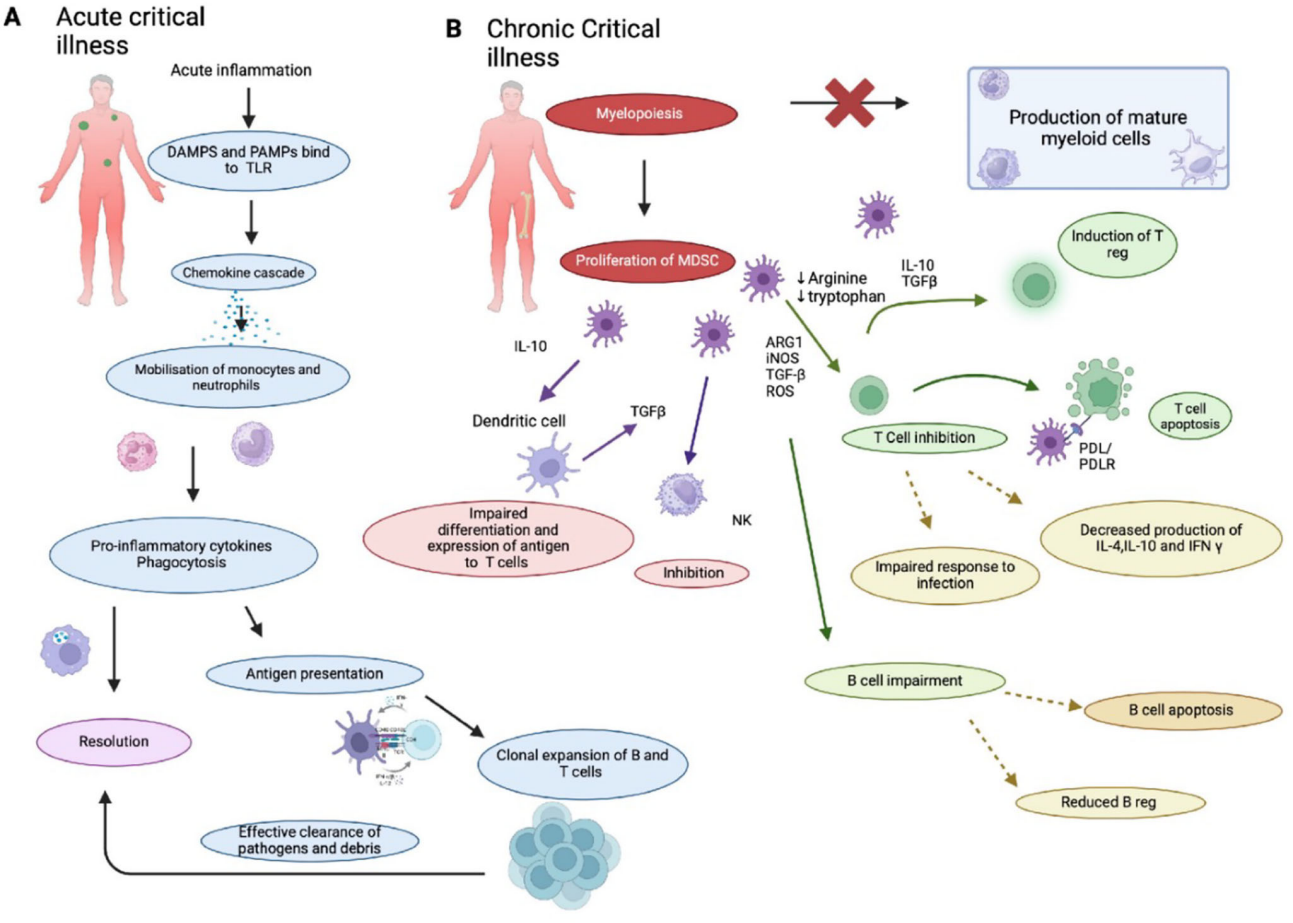
Mechanisms of chronic critical illness. (A) In the acute phase, pathogen‐associated molecular patterns (PAMPs) and damage‐associated molecular patterns (DAMPs) bind to Toll‐like receptors (TLRs), resulting in the expression of several chemokines, causing an egress of peripheral blood mononuclear cells (PBMCs) from the bone marrow and migration to areas of high chemokine concentration. Phagocytosis and production of proinflammatory cytokines and reactive oxygen species (ROS) result in the resolution of the insult. Created with BioRender.com. (B) Myeloid progenitor cells proliferate by releasing immature myeloid cells or myeloid‐derived suppressor cells (MDSC) into the circulation. MDSCs decrease the levels of essential amino acids for T cell function, such as L‐arginine, cysteine, and tryptophan. Increased production of Interleukin (IL)‐10 and transforming growth factor (TGF)‐β results in T cell suppression and induction of regulatory T cells (Tregs). MDSCs express a high level of inducible nitric oxide synthase (iNOS) and produce high quantities of ROS, suppressing T cell function. Natural killer cells (NK) are suppressed via TGF‐β, and dendritic cells via IL‐10. Created with BioRender.com.

***Myeloid‐derived suppressor cells (MDSC)***: In survivors of surgical sepsis are elevated from 4 to 42 days post‐insult compared to healthy controls (**Figure** **3**). The expression of miRNA in patients who rapidly recovered from the septic episode, compared to CCI patients, has the most significant difference in expression, with the genes associated with MDSC being upregulated in the latter (Hollen et al. 2019). A sustained increase in MDSC is related to critical illness, increased nosocomial infections, and poor long‐term functional status (Mathias et al. 2017). 37% of patients die within six months of the initial diagnosis of sepsis. Interestingly, in addition to the rise in acute inflammatory markers, including IL‐6, IL‐8, and IL‐10, these patients had a reduced lymphocyte count at 28 days, with increased markers of catabolism (Stortz et al. 2018).

The CCI phenotype directly affects neurocognitive aging. Sustained MDSC‐mediated immune suppression and persistent senescent secretory‐associated phenotype (SASP) secretion from senescent cells maintain systemic inflammation, linked to BBB disruption, microglial priming, and hippocampal injury in critical illness. Clinical data from the Vanderbilt group found that 26% of respiratory failure or septic shock patients had cognitive scores at 12 months akin to mild Alzheimer's, with severity correlating to ICU brain dysfunction. CCI and maladaptive immune suppression after initial hyperinflammation may foster accelerated neurocognitive aging long after hospital discharge.

**T cells** express the inhibitory checkpoint molecule programmed death‐1 (PD‐1), which is upregulated by the stimulation of T cells. The ligands for this receptor, PD‐L1 and PD‐L2, are expressed on epithelial cells, endothelial cells, macrophages, dendritic cells, and monocytes. In sepsis and trauma, patients who subsequently developed a nosocomial infection or who died had higher PD‐1 expression, increased IL‐10 levels, and a decreased circulating cluster differentiation 4 (CD4) count (Guignant et al. 2011). In a post‐mortem study of the spleen and lungs in septic and non‐septic critically ill patients, splenocytes from septic patients showed a reduced cytokine production at 5 h following stimulation with anti‐CD3/anti‐CD28, less than 10% of that of the control group. Sepsis resulted in an increased expression of PD‐1 on CD4 lymphocytes and increased cytotoxic T‐lymphocyte antigen 4 (CTLA‐4) on CD8 lymphocytes. The expression of the IL‐7 receptor, which is integral in cell survival, decreased in both CD4 and CD8 cells. In the septic lung samples, there was increased expression of PD‐L1 and PD‐L2 on dendritic cells and epithelial cells in the airways (Boomer et al. 2011). In a subsequent study of patients with severe sepsis, blood samples were taken at sepsis onset and one week later. Increased expression of inhibitory cell surface receptors CTLA‐4, T‐cell immunoglobulin and mucin‐domain‐containing‐3 (TIM‐3), and lymphocyte activation gene‐3 (LAG‐3), along with decreased expression of IL‐7, was observed in samples taken 7 days after sepsis onset (Boomer et al. 2012).

### 4.10 Chronic Low‐Grade Inflammation and Neurocognitive Decline

**FIGURE 4:**
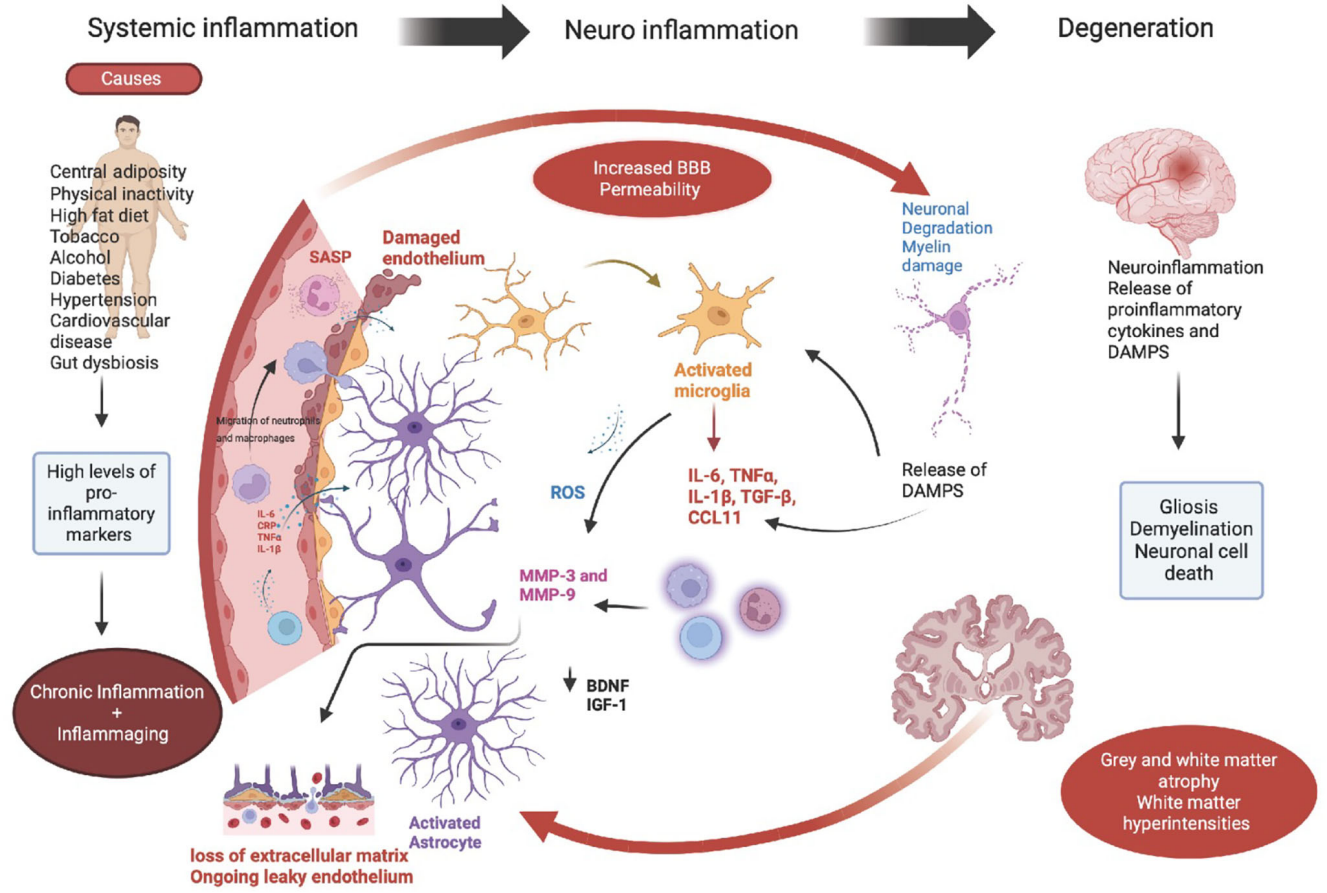
Causes of chronic systemic inflammation in addition to critical illness that predispose individuals to neuroinflammation. Inflammatory changes in the periphery lead to alterations in blood–brain barrier (BBB) permeability that initiates CNS inflammation. Following infiltration of peripheral inflammatory cells, central activation of neuroglia and neuronal damage ensues, resulting in an inflammaged nervous system, loss of gray and white matter and cognitive dysfunction. Abbreviations: SASP – senescent secretory associated phenotype; IL – interleukin; TNF – tumor necrosis factor; TGF – transforming growth factor; CCL – chemokine ligand; ROS—reactive oxygen species; MMP – matrix metalloproteinase; CRP – C‐reactive protein; BDNF – brain‐derived neurotrophic factor; IGF – insulin‐like growth factor; DAMPS – damage‐associated molecular patterns. Created with BioRender.com.

Inflammaging is increasingly recognized as a hallmark of aging (Dugan et al. 2023). Contributing factors include dysfunctional mitochondria, defective autophagy, defective ubiquitination and protein removal, endoplasmic reticulum stress, and activation of the DNA damage response. The accumulation of senescent cells displaying the SASP and age‐associated loss of intestinal barrier permeability results in the translocation of microbial products, driving inflammaging. An increasing number of comorbidities that affect the older population, such as cardiovascular disease, chronic kidney disease, dementia, depression, and diabetes, are recognized because of inflammaging (Conway and A Duggal 2021; Ferrucci and Fabbri 2018; Zhang et al. 2016) (Figure 4).

Inflammation is a significant cause of cognitive decline. The Honolulu‐Asia Ageing study found a strong relationship between the C‐reactive peptide (CRP) concentrations of Japanese American men and the risk of developing vascular and Alzheimer's dementia 25 years later (Schmidt et al. 2002). The Rotterdam Study replicated these results, with elevated plasma levels of IL‐6 and *α*‐1‐antichymotrypsin associated with an increased risk of dementia years later (Engelhart et al. 2004). In the Framingham Offspring study, the inflammatory markers were associated with a greater‐than‐expected loss of total brain volume in older individuals (Jefferson et al. 2007). Central and peripheral inflammation has been observed in patients with Lewy body dementia, and there is a strong association between microglial activation in several brain areas and decreased cognitive scores. Cortical inflammation on PET scans can be detected before the onset of both Alzheimer's and PD (Zhang et al. 2014). However, further research establishing causality and the impact of genetic risk factors, including ApoE and TREM2, for sporadic AD must be established and explored.

High interferon‐gamma (IFN‐γ) levels are associated with poor cognitive performance, suggesting that the duration of inflammation is vital in the pathophysiology of cognitive impairment in sepsis (Calsavara et al. 2018). Serial inflammation measurements and the Confusion Assessment Method for the intensive care unit (CAM‐ICU) are more effective than a single marker or time point measurement of inflammation. No correlation has been shown between a single CAM‐ICU assessment score and long‐term cognitive outcomes (van den Boogaard et al. 2011).

Compared to healthy controls, sepsis patients have fewer naive T cells, more senescent T cells, and fewer recent thymic emigrants. They also show increased immature granulocytes with reduced function and fewer natural killer (NK) cells. A higher neutrophil:lymphocyte ratio correlates with longer critical illness and higher mortality and is also a characteristic of immunosenescence (Duggal et al. 2018).

## 5 Autoimmunity in Critically Ill Patients With Acute Brain Injuries and General Critical Illness With Neurocognitive Deterioration

**FIGURE 5:**
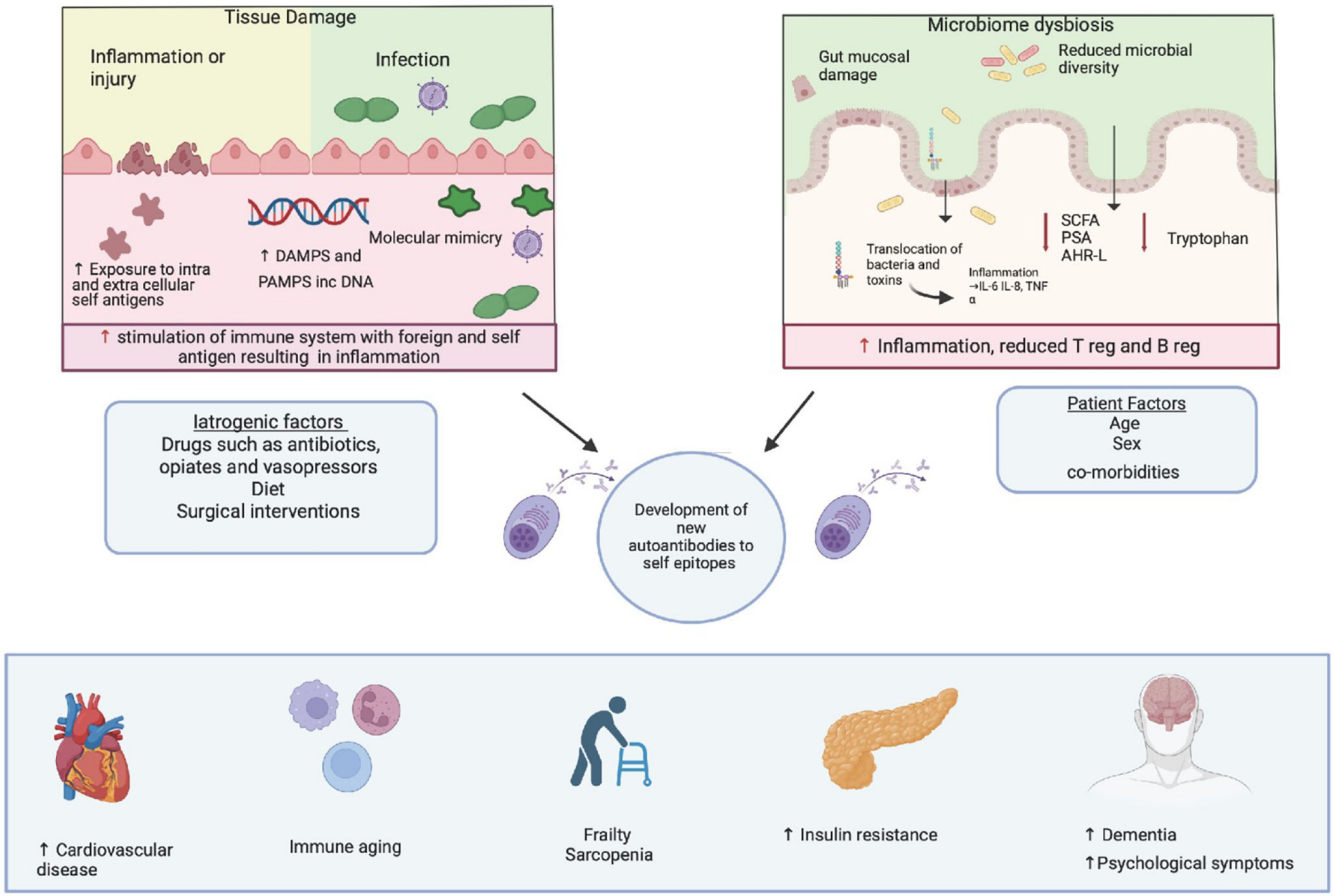
Development of autoimmunity. Critical illness caused by injury, infection, and inflammation leads to tissue damage and exposure of intra‐ and extracellular antigens to the immune system. Damage to gut mucosa because of a combination of ischemia, inflammation, polypharmacy, and dysbiosis results in reduced anti‐inflammatory compounds such as short‐chain fatty acids (SCFA), polysaccharides A (PSA), and aryl hydrocarbon receptor ligands (AHR‐L), and reduced tryptophan resulting in reduced B cell regulation. In addition, translocation of altered gut commensals and toxins can enter the circulation, further stimulating chronic inflammation. Abbreviations: DAMPs – Damage‐associated molecular patterns; PAMPs – pathogen‐associated molecular patterns; DNA – deoxyribonucleic acid; IL – interleukin; TNF – tumor necrosis factor. Created with BioRender.com.

Several mechanisms proposed for autoantibody production include molecular mimicry, bystander activation, viral persistence, the fertile field, and epitope spreading. Molecular mimicry involves shared epitopes between the host and non‐self‐antigens. T cell receptors and B cell immunoglobulins can recognize peptides as pathogenic if they share shape and charge, such as in rheumatic heart disease and group A streptococcus infection (Fujinami et al. 2006). Viral persistence, due to persistent viral infection, stimulates an immune response. Bystander activation results from activated antigen‐presenting cells from an acute infection activating pre‐primed autoreactive T cells. The fertile field can be a mixture of the three mechanisms described above (Fujinami et al. 2006). The production of autoantibodies occurs rapidly in acute illness and remains elevated for weeks after the acute episode (Burbelo et al. 2010) (**Figure** **5**). A key methodological distinction that guides the interpretation of autoantibody research in critical illness is that the presence of an autoantibody in serum or CSF does not confirm it is pathogenic. In autoimmune encephalitis, pathogenicity is indicated when an antibody (i) binds to a known, crucial CNS antigen; (ii) reproduces disease in models or cultured neurons; and (iii) correlates with clinical improvement after immunotherapy that reduces antibody levels (Graus et al. 2016). Most autoantibodies found in acute critical illness do not meet these criteria and likely reflect immune activation triggered by exposure to neural antigens, which may be non‐pathogenic.

Autoimmune disease results from an interplay between B and T cells, with B cells having functions beyond producing autoantibodies. B cells, in addition to their role in producing autoantibodies, act as antigen‐presenting cells and stimulate self‐recognizing T cells. Autoantibodies to neuronal antigens and glia are more frequent in patients with dementia compared to healthy age‐matched controls (Barthel et al. 2023). Autoimmune dementia is associated with antibodies targeting cell surface proteins such as AChR (acetylcholine receptor), CASPR2 (contactin‐associated protein receptor 2), DPPX (dipeptidyl‐peptidase‐like protein), N‐methyl‐D‐aspartate receptor (NMDAR), AMPAR, LGI1, and mGluR5, as well as intracellular proteins such as GFAP, MOG, myelin basic protein (MBP), amphiphysin, Gad 65, Ma, Ri, Yo, and Hu (Gibson et al. 2021; Hansen 2021; Hansen et al. 2021).

### 5.1 Autoimmunity in Brain Trauma

Brain trauma damages the brain's microvasculature and BBB endothelium, releasing CNS components into circulation. Seven days post‐TBI, 25% of patients developed new IgM antibodies, mainly to myelin‐associated glycoprotein (MAG) and MBP, while 65% developed IgG antibodies to TJP1 and MAG. These autoantibodies peaked at 7 days and remained elevated for 6–13 years. Persistent anti‐MAG antibodies correlated with neurofilament light chain (NFL) levels 6–12 months after injury, indicating ongoing neuroinflammation or degeneration (Needham et al. 2021). Patients with a Glasgow Coma Scale (GCS) score of 9–13, 24 h post‐injury have lower anti‐GFAP antibodies, indicating a less severe brain injury. Those with lower GCS scores show higher autoantibody levels and worse six‐month outcomes. Notably, 15.6% of healthy controls also had GFAP autoantibodies, likely due to minor, unreported head injuries (Zhang et al. 2014).

### 5.2 Cerebrovascular Diseases

Stroke induces a rapid proliferation of autoantibodies to CNS antigens in mice with autoantibodies reactive to myelin proteins, NMDAR subunits, and dendritic cytoskeleton proteins present up to 10 days post‐infarct (Ortega et al. 2015). Anti‐NMDAR has been detected within 3 h of ischemic stroke and transient ischemic attack in humans (Dambinova et al. 2003). Anti‐NMDAR autoantibody is associated with increased mortality in sepsis patients (Malfussi et al. 2019). A single stroke increases a person's risk of dementia by twofold, with an initial acute decline followed by a gradual decline over the subsequent 6 years, resulting in significant disability (Levine et al. 2015). The ablation of B cells with anti‐CD20 antibodies has decreased the appearance of delayed cognitive defects in mice (Doyle et al. 2015). In humans, CNS antigens released from injury are thought to first contact B cells draining to the deep cervical lymph nodes and are present within three days of acute injury. However, in mice and post‐mortem studies of patients who have died following stroke, clusters of B cells have been seen in the infarcted area of the brain, and there is evidence of intrathecal production of autoantibodies in humans (Doyle et al. 2015; Prüss et al. 2012). Stroke has been associated with several autoantibodies with targets including NMDAR subunits, GFAP, S100B, MBP, and proteolipid protein (Javidi and Magnus 2019). This broad range of targets means multiple pathways cause cognitive dysfunction and impaired recovery.

### 5.3 General Critical Illness, Without Brain Injury

Meta‐analysis suggests that 36% of patients treated for acute respiratory distress syndrome (ARDS) had cognitive impairment after their critical illness. Older age was a predictive factor for a higher prevalence of cognitive impairment (Pohl et al. 2025). 56% of patients with ARDS and 46% with septic shock rapidly produce at least one high titre of autoantibodies to a range of targets, including aquaporin‐4 and glutamic acid decarboxylase‐65, and non‐CNS antigens such as potassium channel regulator lung protein, gastric ATPase, and IFN‐γ, which peak at 7–14 days and persist at 20 and 28 days (Burbelo et al. 2010). (134)

Coronavirus Disease‐19 (COVID‐19) patients who were mechanically ventilated had more autoantibodies than age‐matched controls (Etter et al. 2022; Bitzogli et al. 2022). Autoantibodies found in these patients include anti‐GAD, anti‐CASPR2, anti‐NMDAR, antibodies to multiple myelin proteins, anti‐yo, anti‐ANA, anti‐dsDNA, anti‐CCP, and anti‐CL IgM (Bitzogli et al. 2022; Franke et al. 2021; Guilmot et al. 2021). The hypothesis that NMDAR antibodies directly cause brain dysfunction in sepsis requires scrutiny. In a prospective study of 101 septic patients, anti‐NMDAR autoantibodies were detected in 28% of cases and predicted hospital mortality but were not associated with brain dysfunction measured by CAM‐ICU (Malfussi et al. 2019). This suggests that in the context of critical illness, NMDAR autoantibodies may serve as a marker of illness severity and neurological injury rather than a direct cause of cognitive deterioration. Autoantibodies to several antigens have been found in COVID‐19 and respiratory viral infections such as the flu, with antibodies to myelin and lung surfactant proteins more frequent in those with more severe disease. Notably, these individuals are also reported to exhibit a greater frequency of antibody generation, elevations in inflammatory cytokines (IL‐6, IL‐1β, and TNFα), and higher brain injury markers (NFL, tau, and GFAP) in the acute and convalescent periods (Needham et al. 2022).

High tissue protein antibodies correlated with autoantibodies, tissue antigens, and inflammatory markers like CRP, ferritin, and lactate. Patients with autoantibodies against hypocretin receptor 2, found in the hypothalamus, had low GCS. In a study comparing critical illness patients with and without COVID‐19, 20% of non‐COVID‐19 and 30% of COVID‐19 patients had autoantibodies targeting various antigens. Critically ill COVID‐19 patients showed specific brainstem epitope antibodies compared to less severe cases (Lucchese et al. 2022).

CSF of patients with COVID‐19 shows no SARS‐CoV‐2 immunoglobulins in the CNS. Although 80% had elevated CSF IgG levels, evidence suggests these were transferred via a leaky BBB rather than produced locally (Jarius et al. 2022). High CSF protein, glucose, and lactate levels, as well as intrathecal detection of peripherally produced antibody levels, have been linked to severe COVID‐19 infection and critical illness (Etter et al. 2022). IL‐8, vascular endothelial growth factor‐A (VEGF‐A), and receptor for advanced glycation end products have been implicated in BBB disruption, which could be responsible for the ingress of autoantibodies into the CNS. Detection of the COVID‐19 virus by PCR in the CSF in COVID‐19‐infected patients is often absent despite systemic infection and neurological symptoms (Schaller et al. 2020). Senescence prevents the spread of damaged cells, reducing cancer risk (Behfar et al. 2022; Gorgoulis et al. 2019; Kirkland and Tchkonia 2020); some senescent cells develop a senescence‐associated secretory phenotype, comprising between 30% and 70% of the cell population (**Table** **4**) (Kirkland and Tchkonia 2020; Behfar et al. 2022; Chaib et al. 2022; Gorgoulis et al. 2019).

**TABLE 4:** Known senescence‐associated secretory phenotype factors.

Soluble signaling factors	IL‐6, IL‐1, IL‐8, IL‐13, IL‐15, GM‐CSF, IGF, MCP‐1, VEGF‐A, TNF‐α, TGFβ, IFN γ
Secreted proteases	MMP‐1, MMP‐3, MMP‐10, uPA, SERPINS, TIMPS, tPA, PAI‐1, PAI‐2
Extracellular matrix proteins and insoluble proteins	Fibronectin, collagens, laminin
Bioactive lipids	Saturated fatty acids, bradykinins, prostanoids, ceramides
Noncoding nucleotides	Mitochondrial DNA, miRNAs, circular DNAs
Non‐protein molecules	PGEs, nitric oxide, ROS

Abbreviations: IL, interleukin; GM‐CSF, granulocyte‐macrophage colony‐stimulating factor; IGF, insulin‐like growth factor; MCP‐1, monocyte chemoattractant protein‐1; VEGF‐A, vascular endothelial growth factor A; TGFβ, transforming growth factor‐β; IFN‐g, interferon‐g; MMP, matrix metalloproteinase; uPA, urokinase‐type plasminogen activator; SERPINs, serine protease inhibitors; TIMPs, tissue inhibitors of metalloproteinases; tPA, tissue plasminogen activator; PAI, plasminogen activator inhibitor; DNA, deoxyribonucleic acid; miRNAs, micro‐ribonucleic acids; PGE, prostaglandin E; ROS, reactive oxygen species.

Increasing evidence shows that acute conditions activate senescence pathways, which are beneficial in the short term but cause chronic inflammation and aging in the long term. Routine mechanical positive‐pressure ventilation can induce senescence markers, including increased p53‐associated p21Cip1 and heterochromatin foci (Blázquez‐Prieto et al. 2021). COVID‐19 has also been shown to trigger senescence not only in the immune system and the lungs, with upregulation of p53, p21Cip1, IL‐6, and p16Ink4a, but also in epithelial and endothelial cells, making them more prone to senescence, with cognitive impairment, neuroinflammation, and poor cognitive scores 6 years after illness for reasons that are not yet clear (Wang et al. 2021; Bassi et al. 2021).

Studies of older hospitalized patients found polypharmacy linked to dysbiosis, with proton pump inhibitors, antidepressants, and antipsychotics most associated with microbiome changes (Ticinesi et al. 2017). Antibiotics reduce microbiome diversity, with incomplete recovery post‐administration, with implications for patients treated for infections in an acute illness (Dethlefsen and Relman 2011; Szychowiak et al. 2022). Loss of microbial diversity alters the gut's mucosal lining, increasing the translocation of microbial toxins and contributing to chronic inflammation or inflammaging. Dysbiosis also impairs mucosal immunity, particularly Peyer patches, reducing IgA production (Szychowiak et al. 2022; Gatt et al. 2007).

### 5.4 Potential Targets and Unresolved Questions

Future ASI research should explore why some experience transient effects while others develop chronic deficits and how to prevent this. Understanding glial cells' roles and modulating their states is vital. The long‐term impact of mild episodes, especially in vulnerable groups, warrants study. Developing biomarkers for risk prediction, neuroinflammation monitoring, and treatment evaluation is urgent. Investigating endogenous inflammation‐resolution pathways, like SPMs, and their therapeutic potential is essential. Research on how systemic factors, such as metabolic issues and the gut microbiome, influence brain health and cognition is also necessary.

## 6 Potential Therapeutic Targets and Strategies

There is a growing understanding of the mechanisms linking ASI to cognitive impairment, but evidence‐based treatments remain elusive.

*Broad anti‐inflammatory drugs*: While NSAIDs and corticosteroids have been considered, their use is complicated by potential side effects, the need for optimal timing, and concerns about nonspecific immunosuppression.

Rapamycin (clinically known as sirolimus) is a macrolide immunosuppressant used to prevent organ rejection in renal transplantation and to treat sporadic lymphangiomyomatosis. This rare multi‐system disorder causes respiratory impairment (Compendium E.M. n.d.). Rapamycin exerts its effects by inhibiting mammalian target of rapamycin (mTOR)1 and mTOR2. mTOR1 is sensitive to rapamycin and controls autophagy, protein translation, and the phosphorylation of S6K (ribosomal biogenesis), 4E‐BP1 (translation), and ULK‐1 (autophagy). Early studies suggest it may improve longevity, coordination, and muscle strength; alleviate AD‐induced cognitive impairment in animal studies; and reduce age‐related pathologies such as adiposity, liver steatosis, and reduced mobility (Apelo and Lamming 2016; Wang et al. 2021). Rapamycin has side effects like impaired wound healing, fluid buildup, high cholesterol, blood issues, infection risk, metabolic problems, liver abnormalities, and gastrointestinal upset.

*Specific cytokine antagonists*: Therapies targeting cytokines like anti‐TNF‐*α* biologics show promise in preclinical and some clinical conditions, but more evidence is needed on their efficacy and safety for preventing ASI‐induced cognitive decline, especially regarding CNS penetration and systemic immune effects.

Sitagliptin, a DPP‐4 inhibitor, improves cognitive function in elderly diabetic patients by improving neuronal insulin receptor and mitochondrial function and reducing oxidative stress (Isik et al. 2017). In stroke, hyperglycemia aggravates injury from ischemia, enlarging the infarct size and worsening the outcome. Sitagliptin has been found to suppress nuclear factor kappa (NF‐κ), resulting in decreased inflammatory mediators such as TNF‐*α* and IL‐6 and elevation of anti‐inflammatory cytokine IL‐10. In addition, it has antioxidants, is anti‐apoptotic, and suppresses the activation of NMDA receptors (El‐Sahar et al. 2015). The brain is a highly metabolic organ with a high oxygen turnover and susceptibility to oxidative stress.

*BBB protection/restoration*: Strategies aimed at stabilizing and preserving BBB integrity or promoting its repair could limit the entry of peripheral inflammatory mediators into the CNS (161).

*Modulation of microglial activation*: Shifting microglia from a pro‐inflammatory to a neuroprotective/reparative phenotype is an attractive, albeit complex, therapeutic goal.

*Antioxidant therapies*: Antioxidants have been explored, but clinical trial results have often been disappointing, possibly due to issues with potency, delivery, and timing. The brain is a highly metabolic organ with a high oxygen turnover and susceptibility to oxidative stress. The ratio of oxidants to antioxidants, such as vitamin E (*α*‐tocopherol), must be maintained to prevent oxidative damage to proteins and DNA. Vitamin E has been studied, as it is lipid‐soluble and, as such, can cross the BBB; however, no studies thus far have shown any change in the progression of cognitive impairment (Farina et al. 2017).

*Vagus nerve stimulation*: Electrical stimulation of the vagus nerve can attenuate ASI (the “inflammatory reflex”) and has shown some promise in preclinical models of neuroinflammation.

*Targeting inflammation resolution*: Promoting endogenous resolution pathways, for example, through administering SPMs or their precursors, offers a novel approach to resolving inflammation rather than actively suppressing its initiation. Significant challenges remain, such as identifying the optimal therapeutic window for intervention, drug penetration across the BBB, target specificity for immunosuppression with interindividual variability, and requiring precision medicine approaches. The brain's vulnerability to systemic disturbances highlights the need for disciplinary research bridging immunology and neuroscience. This research should focus on actively pursuing innovative strategies, such as promoting early inflammation resolution.

Resveratrol (3,5,4′‐trihydroxystilbene), an agent abundant in the skin of grapes and red wine, has been shown to promote longevity in several species, but often in high‐fat, high‐sugar diets. In addition, it is less effective than rapamycin and, at cytostatic and near‐toxic concentrations, can prevent cellular senescence (Demidenko and Blagosklonny 2009). Resveratrol activates Sirtuins‐1, 3 and 5; however, most of the effects on preventing neurodegeneration have been attributed to SIRT 1. In mouse embryo models of Huntington's disease, resveratrol restored mitochondrial function with enhanced expression of electron transporter genes with improved motor coordination and learning (Naia et al. 2017). In murine stroke models, it attenuated the increase in epithelial and vascular permeability of the small intestine induced by cerebral ischemia, resulting in reduced inflammation and altered balance of T17/Treg toward Treg and Th1/Th2 ratio toward Th2 (Zhao et al. 2023).

*Calorie or dietary restriction (DR) or mimics*: 50% DR has been shown to extend the life span and reduce age‐related chronic diseases in various organisms such as yeast, worms, and rats (Bordone and Guarente 2005). Biosphere experiments and CALERIE (Comprehensive Assessment of Long‐Term Effects of Reducing Intake of Energy) studies have attempted to reduce calorie intake while maintaining micronutrient content (Dorling et al. 2021; Walford et al. 2002). They have been associated with improved cardiovascular outcomes and decreased inflammation, but problems include adherence, lack of satiety, loss of libido, and decreased bone health (Ingram and Roth 2015). Bariatric surgery, acarbose, and orlistat impair nutrient absorption in patients but are unsuitable for this patient population (Ingram and Roth 2015).

Given the lack of evidence and logistical difficulties, there has been interest in studying downstream signaling due to calorie restriction. AMP‐activated protein kinase (AMPK), sirtuins, and mTOR are critical mechanistic pathways in preventing aging. Many of the dietary restriction mimetics interact with these cellular pathways. mTOR is divided into mTOR1 (sensitive to rapamycin) and mTOR2 (not sensitive to rapamycin) (Sarbassov et al. 2004). mTOR1 responds to the extracellular environment through the availability of glucose, amino acids, hormones, growth factors, and hypoxia. When mTOR1 is activated, there is increased protein, lipid, and nucleotide synthesis; decreased autophagy and mitophagy; and increased aging (Mannick and Lamming 2023). mTOR regulates mitochondrial function by stimulating the production of messenger ribonucleic acid (mRNA) in the nucleus for various enzymes involved in mitochondrial respiration and adenosine triphosphate (ATP) production (Morita et al. 2015). This increases energy production and meets the high demands of senescent and cancer cells (Frasca et al. 2021). mTOR function is linked to neurodegeneration because Alzheimer's dementia, PD, Lewy body dementia, and Huntington's disease are all related to protein aggregation (Papadopoli et al. 2019). mTOR regulates autophagy and the clearance of misfolded proteins, which are the pathological trademarks of these conditions.

Sirtuins 1–7 are present in yeasts, model organisms, and humans, and the system's complexity increases with the complexity of the animal. In mammals, the various Sirtuins are localized to specific parts of the cell; SIRT 1 and 2 are in the cytoplasm; 3, 4, and 5 are in the mitochondria; and 6 and 7 are in the nucleus. Sirtuin 1 deletion in mouse models of Huntington's exacerbates the toxicity associated with aberrant accumulation of proteins and, in AD, significantly increases *β*‐amyloid plaques and related inflammation. It deacetylates various proteins, including those associated with DNA repair and autophagy, and inhibits mTOR1. SIRT 3 and 5 are mitochondrial and decrease oxidative stress; SIRT 3 facilitates gluconeogenesis from amino acids, and its expression is increased in tissues that are highly metabolically active, such as brown adipose tissue, heart, liver, and brain; both 3 and 5 have been implicated in insulin production and sensitivity (Zhao et al. 2020).

Metformin has been extensively studied in the context of aging in recent years; however, the mechanisms by which it exerts its effect are complex. A systematic review suggested that metformin leads to a reduction in all‐cause mortality in non‐diabetics and diabetics (Campbell et al. 2017). Metformin interacts with mitochondrial complex 1, reducing oxidative phosphorylation and the ATP to AMP ratio, activating AMPK and inhibiting mTOR1 (Mohammed et al. 2021). The role of metformin specifically in neurodegeneration is not fully established, but it is anti‐inflammatory by inducing SIRT‐1 expression, inactivating Nf‐kB, and reducing p65 acetylation in peripheral blood mononuclear cells (Xu et al. 2015). Metformin usage enhanced the antibody response to influenza vaccination in elderly individuals with diabetes to the levels observed in young, healthy adults. B cells from elderly patients with T2DM are proinflammatory, hypermetabolic, and senescent. However, metformin reduced IL‐6 and TNF‐α mRNA expression, decreased glucose uptake, and reduced mRNA expression of glucose transporter‐1 and metabolic enzymes involved in oxidative phosphorylation. In addition, it decreased proinflammatory double‐negative (CD27‐IgD‐) ABC and decreased autoimmune antibody secretion (Frasca et al. 2021). Metformin has shown promise in restoring endothelial function after injury, such as hypoxia and distension from mechanical ventilation, which are features of critical illness and its management (Tsaknis et al. 2012). However, metformin is not without its side effects, including digestive issues, headaches, and a vitamin B12 deficiency.

*GLP‐1 receptor agonists*: Glucagon‐like peptide‐1 receptor agonists (GLP‐1RAs), such as liraglutide and semaglutide, have potential neuroprotective effects in neuroinflammatory states (Monti et al. 2022). GLP‐1 receptors are expressed in hippocampal neurons, cortical cells, and glial populations. GLP‐1R signaling exerts direct anti‐inflammatory effects, primarily by inhibiting the NF‐κB pathway and attenuating microglial activation. In addition, GLP‐1RAs reduce neuronal oxidative stress, promote BDNF‐mediated neurogenesis, improve cerebral insulin signaling, and attenuate BBB disruption in preclinical models of systemic inflammation and neurodegeneration (Spezani and Mandarim‐de‐Lacerda 2025).

In preclinical sepsis models, liraglutide has been shown to reduce hippocampal neuronal apoptosis, attenuate microglial activation, and improve spatial memory performance (Li et al. 2026). Clinically, cardiovascular outcomes trials have generated signals toward preserved cognitive function and reduced dementia risk in patients treated with semaglutide and liraglutide, although these data derive from metabolically ill rather than critically ill populations, and the cognitive effects may be partially mediated by cardiovascular risk reduction rather than direct neurological action (De Giorgi et al. 2025). A key translational challenge for GLP‐1RAs is the limited penetration through the BBB due to their molecular size. Nanoparticle‐based delivery systems, including lipid nanoparticles, polymeric poly(lactic‐co‐glycolic acid) (PLGA) nanoparticles, and exosome‐mediated delivery, are under active preclinical investigation as strategies to improve CNS bioavailability (Zeng et al. 2025).

GLP‐1 receptor agonists after critical illness require careful evaluation of their musculoskeletal risks. Major clinical trials like STEP, SCALE, and SURMOUNT show that while these drugs lead to significant weight loss, 15%–40% of it is lean muscle mass loss (Karakasis et al. 2025). This is concerning for ICU survivors who already face sarcopenia and ICU‐acquired weakness due to critical illness's catabolic effects, prolonged immobility, and PICS‐related inflammation and muscle breakdown (Puthucheary et al. 2013). In older or frail patients, further muscle loss from these drugs may worsen existing musculoskeletal vulnerability, increasing fall risk, functional decline, and mortality, counteracting post‐ICU rehab goals. A study on older adults with type 2 diabetes found that semaglutide accelerated sarcopenia over 24 months, reducing muscle mass and grip strength (Ren et al. 2025). Strategies to mitigate this include resistance exercise, adequate dietary protein (≥30–35 g per meal), and possibly lower‐potency or shorter treatment courses for frail or ICU‐weak patients. Future research should include body composition, muscle strength, and functional outcomes alongside cognitive and metabolic measures.

*Senolytic drugs*: Senescence contributes to age‐related disorders, and senolytics are being tested following the finding that calorie restriction reduced senescent cells in mice. They are categorized by generation: first‐generation senolytics, based on hypothesis‐driven mechanisms, include agents listed in Table 5 (Chung and Kim 2022; Fuhrmann‐Stroissnigg et al. 2017; Gonzales et al. 2022; Le et al. 2021; Li et al. 2019; Liu et al. 2018; Zhu et al. 2021; Zhu et al. 2017; Zhu et al. 2016). Second‐generation agents, developed via high‐throughput screening, include nanoparticles and vaccines. Senolytics have limitations, such as challenges in clinical translation, safety concerns, and limited research on their use in neurological senescent cells. The ideal senolytic would target senescent cells precisely without off‐target effects. Navitoclax (ABT263) hasn't been used outside animals due to side effects like thrombocytopenia and neutropenia and its ineffectiveness in adipose tissue, which is rich in senescent cells in the aged (Zhu et al. 2016). Dasatinib, a tyrosine kinase inhibitor for leukemia that disrupts anti‐apoptotic pathways in senescent cells, has been combined with quercetin to treat diabetic kidney disease. It reduces senescent cells in adipose tissue and lowers plasma IL‐6 and IL‐2 levels, part of the SASP profile (Hickson et al. 2019). An open‐label pilot study in patients with idiopathic pulmonary fibrosis using dasatinib and quercetin found significant functional improvements and was well‐tolerated. A double‐blind, placebo‐controlled trial is currently underway and recruiting. Ongoing trials also examine dasatinib and quercetin for age‐related osteoporosis, relatives of type 2 diabetes patients, childhood cancer survivors, and hematopoietic stem cell transplant survivors (Kirkland and Tchkonia 2020).

**TABLE 5:** Senolytic drugs.

Agent	Mechanism of action
Dasatinib	Tyrosine kinase inhibitor that inhibits SRc kinase and promotes apoptosis
Quercetin	A flavinoid that inhibits BCL‐2
Fisetin	A flavinoid that inhibits BCL‐2
Luteolin	Inhibits oxidative stress‐induced cellular senescence via SIRT 1 and p53
Curcumin and curcumin analog	BCL‐2 inhibitor
Navitoclax (ABT263)	BCL‐2 inhibitor
FOXO4 related peptide	Activates TP53‐mediated apoptosis
Nutilin 3a	MDM2/p53 inhibitor
A1331852	BCL‐2 inhibitor
A1155463	BCL‐2 inhibitor
Geldanamycin	HSP90 inhibitor that triggers apoptosis by destabilizing AKT
Tanespimycin	HSP90 inhibitor that triggers apoptosis by destabilizing AKT
Alvespimycin	HSP90 inhibitor that triggers apoptosis by destabilizing AKT
Piperlongumine	Induction of oxidation resistance 1 (OXR1) degradation

Animal models show that dasatinib and quercetin reduce NFT, ventricular enlargement, and clear senescent cells. The SToMP‐AD pilot study investigates CNS penetration and effects on physical and cognitive function, serving as a proof of concept for phase 2 trials (Gonzales et al. 2022).

### 6.1 The Role of Non‐Pharmacological Interventions

There is growing evidence that non‐pharmacological interventions, including exercise, social therapy, and cognitive training, can have a key role to play as a potential therapy in long‐term cognitive impairment. A 2023 systematic review and meta‐analysisfound that non‐pharmacological interventions were beneficial in elderly populations with dementia. These non‐pharmacological interventions offer a significantly lower side‐effect burden than pharmacological interventions. However, further studies would be very beneficial for determining the efficacy and optimal format of non‐pharmacological interventions.

Physical exercise and structured rehabilitation represent a mechanistically compelling neuroprotective intervention. Exercise has pleiotropic effects underlying neurocognitive decline. It promotes hippocampal neurogenesis and synaptic remodeling, stimulates mitochondrial biogenesis via AMPK/PGC‐1*α* signaling, enhances cerebrovascular function through angiogenesis and NO‐dependent vasodilation, and reduces systemic inflammation through the downregulation of NF‐κB signaling and the release of anti‐inflammatory myokines (Kong et al. 2025). A central mediator of these effects is BDNF, which is upregulated after aerobic exercise and promotes neuronal survival, synaptic plasticity, and hippocampal neurogenesis, processes that are suppressed by the neuroinflammation in acute critical illness (Liu and Nusslock 2018). Skeletal muscle contraction also drives the systemic release of irisin (via FNDC5/irisin cleavage) and other myokines, including IL‐6, which cross the blood–brain barrier and initiate neuroprotective effects in hippocampal circuits (Lourenco et al. 2019).

Critically, these mechanisms are now supported by clinical evidence directly applicable to the ICU survivor population. A landmark randomized controlled trial by Patel et al. (2023) demonstrated that early mobilization in mechanically ventilated patients reduced the incidence of cognitive impairment at one year by approximately 50%, representing the first prospective RCT evidence of a non‐pharmacological intervention reducing long‐term neurocognitive sequelae of critical illness. Future clinical trials in this population should incorporate neuroimaging, BDNF, and validated cognitive endpoints as co‐primary outcomes alongside physical function measures.

## 7 Future Directions

Despite advances in understanding post‐illness cognitive decline, key questions remain. First, mechanistic stratification is urgent; current evidence can't fully determine whether critical illness accelerates pre‐existing inflammaging or triggers independent neuroimmune pathways, which likely vary with age, frailty, and biological reserve. Biomarker‐based endotyping at ICU admission, using epigenetic age, microglial markers, and inflammaging burden, could enable targeted neuroprotection, with longitudinal studies needed to link accelerated biological aging to long‐term cognition. Second, neurotherapeutic targets need clinical trials. Agents such as senolytics, GLP‐1 receptor agonists, mTOR inhibitors, and metformin show promise in preclinical studies, but no large Phase III trials have been conducted for post‐critical‐illness cognitive decline. Adaptive trials with biomarkers and neuroimaging could fast‐track this. Third, research on rehabilitation programs for neuroprotection is necessary. While ICU mobilization shows cognitive benefits, the optimal timing, intensity, and combination of physical and cognitive rehabilitation remain unclear. Trials with neuroimaging, BDNF, and cognitive testing are needed. Fourth, the impact of systemic therapies on musculoskeletal health in frail, post‐illness patients requires careful study. Older adults on prolonged GLP‐1 therapy face sarcopenia risk; future research should include body composition, strength, and mobility outcomes.

Fifth, the gut–brain axis and microbiome interventions are underexplored. Post‐illness dysbiosis impairs short‐chain fatty acid production and may promote neuroinflammation; trials of pre‐ and probiotics are lacking. Finally, equitable access to neuroprotective treatments is crucial. Post‐ICU follow‐up, rehabilitation, and drugs are unevenly distributed globally and socioeconomically. Research should include implementation to ensure benefits reach all patients.

## 8 Conclusion

Acute and long‐term cognitive impairment after critical illness results from multimodal causes. The initial inflammatory and immune responses, vital for survival, affect more than 80% of cellular pathways in leukocytes during acute diseases such as trauma, burns, and exposure to bacterial endotoxin. Within 12 h, innate immunity, inflammation, and pattern‐recognition genes like TLRs increase, while antigen‐presenting and T‐cell activation genes decrease. Patients who rapidly recover return to baseline gene regulation, whereas chronically ill patients experience ongoing inflammation and immune dysregulation. Chronic low‐grade inflammation, common in aging, links to conditions causing cognitive decline and dementia, with immune aging and increased autoimmunity. Autoantibodies to neuroglia are more frequent in dementia, indicating T cell‐dependent autoimmunity. Long‐term ASI inflammation from critical illness disrupts physiology, influencing microbiome health, aging, and organ dysfunction.

Exposure to CNS antigens in the presence of persistent inflammation leads to autoimmunity and organ dysfunction. Addressing post‐acute neurocognitive decline is societally crucial. Future research should explore autoantibodies from critical illness‐related neurodegeneration, as they can be measured and may signal damaging inflammation. Identifying damage allows treatment adjustments to improve quality of life. Ongoing research also targets senescent cells, with promising therapies for age‐related diseases.

## Nomenclature

AChR: acetylcholine receptor
AD: Alzheimer's disease
AMPK: AMP‐activated protein kinase
ARDS: acute respiratory distress syndrome
ASI: acute systemic inflammation
ATP: adenosine triphosphate
BBB: blood–brain barrier
BDNF: brain‐derived neurotrophic factor
CAM‐ICU: confusion assessment method for the intensive care unit
CASPR2: contactin‐associated protein receptor 2
CCI: chronic critical illness
CD4: cluster differentiation 4
CNS: central nervous system
COVID‐19: Coronavirus Disease‐19
CRP: C‐reactive peptide
CSF: cerebrospinal fluid
CTLA‐4: cytotoxic T‐lymphocyte antigen
DNA: deoxyribonucleic acid
DPPX: dipeptidyl‐peptidase‐like protein
GAD65: glutamic acid decarboxylase‐65
GCS: Glasgow Coma Scale
GFAP: glial fibrillary acidic protein
IFN‐γ: interferon‐gamma
IL‐10: interleukin‐10
IL‐1beta: interleukin‐1β
IL‐6: interleukin‐6
IL‐8: interleukin‐8
LTP: long‐term potentiation
MAG: myelin associated glycoprotein
MBP: myelin basic protein
MDSC: myeloid‐derived suppressor cells
mRNA: messenger ribonucleic acid
mTOR: mammalian target of rapamycin
NFL: neurofilament light chain
NF‐κ: nuclear factor kappa
NK: natural killer cells
NO: nitric oxide
NTS: nucleus tractus solitarius (NTS)
PD: Parkinson's disease
PD‐1: programmed death‐1
PFC: prefrontal cortex
PICS: post‐intensive care syndrome
POCD: post‐operative cognitive dysfunction
RNS: reactive nitrogen species
ROS: reactive oxygen species
SASP: senescent secretory‐associated phenotype
TNF‐alpha: tumor necrosis factor‐α
VEGF‐A: vascular endothelial growth factor‐A

## Author Contributions

**Errin Lawrence**: conceptualization, writing – original draft, investigation, data curation, visualization. **Daniel Fulton**: conceptualization, supervision, writing – review and editing. **Poppy Brown**: investigation, writing – review and editing, methodology, data curation. **Subashini Suresh**: data curation, writing – review and editing, validation. **Mark Morris**: writing – review and editing, validation. **Paraskevi Goggolidou**: validation, writing – review and editing. **Sree Chaithanya**: writing – review and editing, validation. **Marcus Abbawy**: validation, writing – review and editing. **Amelia Wild**: investigation, writing – original draft, data curation. **Prashant Nasa**: validation, writing – review and editing. **Niharika A. Duggal**: supervision, writing – review and editing, conceptualization. **Fang Gao‐Smith**: writing – review and editing, validation, supervision. **Zubair Ahmed**: conceptualization, writing – original draft, writing – review and editing, validation, supervision. **Suresh Renukappa**: writing – review and editing, validation. **Tonny Veenith**: conceptualization, writing – review and editing, writing – original draft, supervision, project administration.

## Funding

The authors have nothing to report.

## Ethics Statement

Ethical review was not required as this is a review article of published studies and does not report primary data involving humans or animals.

## Conflicts of Interest

The authors declare no conflicts of interest.

## Data Availability

No primary data were generated as part of this study, as it is a review of published works.
